# An Arduino-Based Resonant Cradle Design with Infant Cries Recognition

**DOI:** 10.3390/s150818934

**Published:** 2015-08-03

**Authors:** Chun-Tang Chao, Chia-Wei Wang, Juing-Shian Chiou, Chi-Jo Wang

**Affiliations:** Department of Electrical Engineering, Southern Taiwan University of Science and Technology, 1, Nan-Tai St., Yongkang District, Tainan City 71005, Taiwan; E-Mails: tang@mail.stust.edu.tw (C.-T.C.); 4992c015@stust.edu.tw (C.-W.W.); chijo@mail.stust.edu.tw (C.-J.W.)

**Keywords:** Arduino, resonance, cradle, infant cry, fundamental frequency

## Abstract

This paper proposes a resonant electric cradle design with infant cries recognition, employing an Arduino UNO as the core processor. For most commercially available electric cradles, the drive motor is closely combined with the bearing on the top, resulting in a lot of energy consumption. In this proposal, a ball bearing design was adopted and the driving force is under the cradle to increase the distance from the object to fulcrum and torque. The sensors are designed to detect the oscillation state, and then the force is driven at the critical time to achieve the maximum output response while saving energy according to the principle of resonance. As for the driving forces, the winding power and motors are carefully placed under the cradle. The sensors, including the three-axis accelerometer and infrared sensor, are tested and applied under swinging amplitude control. In addition, infant cry recognition technology was incorporated in the design to further develop its functionality, which is a rare feature in this kind of hardware. The proposed nonlinear operator of fundamental frequency (f0) analysis is able to identify different types of infant cries. In conclusion, this paper proposes an energy-saving electric cradle with infant cries recognition and the experimental results demonstrate the effectiveness of this approach.

## 1. Introduction

Enhancing sleep quality is an important research topic, as quality sleep is important for everyone, especially for infants [1,2]. A comfortable electric cradle with a low power consumption that can let infants fall asleep quickly is desired by many parents [3] and numerous novel inventions based on swing mechanisms in the form of springs or rods have been developed [4,5]. For engineers, the ideal oscillation occurs in an undamped system where the system oscillates at a natural frequency with no energy loss. Unfortunately, no such product exists at the present time [6], as most electric baby swing products have the drive motor tightly linked with the bearings leading to an overdamped system with high power consumption. 

The motivation to develop a resonant cradle system comes from Wang *et al*. [7,8], who were highly acclaimed for finding the effect of resonance on blood pressure to verify the corresponding Chinese medicine theory. They found that each organ has its own resonance frequency, and due to this, a human’s heart only needs to consume a minimal amount of power. 

Firstly, in this design, a ball bearing is adopted to yield an underdamped system and the driving force is placed under the cradle to increase the distance from object to fulcrum and torque. Once the driving force is synchronized with the cradle’s vibration, the maximum output response will be achieved according to the principle of resonance. In practical situations, the oscillation frequency of a cradle will vary depending on the weight of the baby, so a driving force with fixed frequency is not capable of achieving the lowest possible power consumption. To achieve this, it needs the help of appropriate sensors to detect the oscillation state, and then the force will be driven during the critical time to save energy and control the swinging amplitude.

Arduino is an open-source electronics platform based on easy-to-use hardware and software launched in 2005 [9]. The goal of Arduino is to create control devices for projects that are easy to use and inexpensive to acquire [10], and this is also consistent with the requirements of this proposed system. Arduino UNO, a microprocessor board with Atmega328 with 32 KB memory, 2 KB of RAM and 1 KB EEPROM, was utilized to implement the proposed design.

In addition to the power consumption problem, the timing of the cradle operation is also an important issue. It is not appropriate for an electric cradle to be always in a swinging state because an intelligent electric cradle should be able to stop periodically and swing autonomously when infant cries are detected. For newborn infants, crying is the primary way for them to express their needs or feelings such as hunger, sleepiness, pain etc., all of which differ in terms of duration and frequency. These audio features across time and frequency domains can be extracted for acoustical analysis by popular feature extraction methods such as Linear Prediction Coefficients (LPC) [11] and Zero Crossing Rates (ZCR) [12]. In addition, fundamental frequency (f0), the median frequency of vocal fold vibration which is often referred to as voice pitch [13], is often used for acoustical analysis. To increase the accuracy of the infant cries identification, classifiers with learning capabilities, such as Support Vector Machine (SVM) [14] or Support Vector Regression [15] are also applied. 

In previous academic research, hardware realization and real-time analysis is not considered but in this paper the fundamental frequency analysis is adopted to recognize a baby’s hunger and pain cries in real-time. Previous research data has shown no significant difference in fundamental frequency due to the sex or age of an infant [16]. A nonlinear operator of fundamental frequency analysis is proposed, which is expected to provide a simple yet effective implementation for infant cries recognition.

The rest of the paper is organized as follows: Section 2 presents the resonant cradle design. In Section 3, the method for infant cries recognition is provided. Finally, Section 4 concludes the final design and provides proposals for future work.

## 2. Resonant Cradle Design

**Figure 1:**
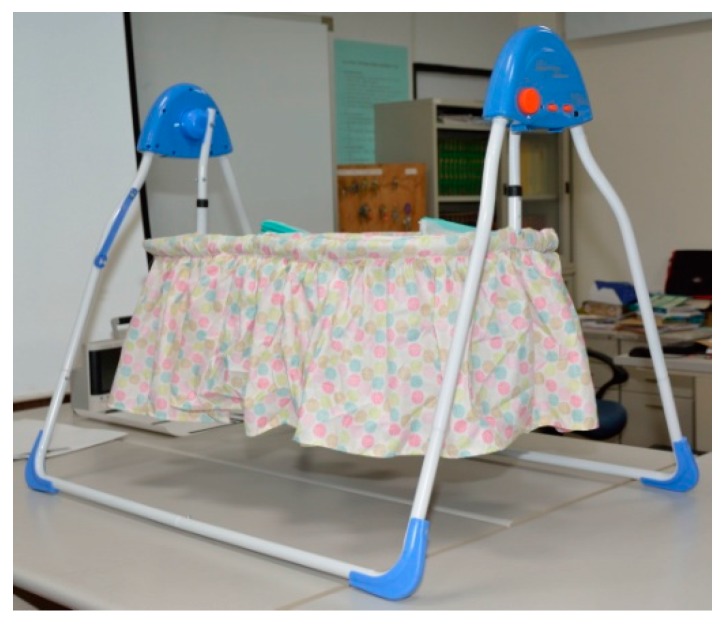
A commercial cradle design example.

Traditional cradles without electricity can be easily lifted by rope and driven by human hands. However, many modern electric cradles cannot swing freely since the driving motor is tightly linked to the bearing. The nature of this overdamped system consumes a considerable amount of power. Figure 1 shows a commercial cradle design example [17], which is difficult to move manually without electricity. If the user pulls the cradle to about a 15° swing angle and releases it, the cradle will come to a complete halt in about 5 s.

For an undamped system, if the driving force has the same frequency as the systems natural frequency, pure resonance will occur and the system output will finally reach infinity. However, in reality, air resistance causes energy loss, thus no truly undamped system actually exists. An underdamped system with a small damping ratio is desired for a practical oscillation system design. 

The simplified pendulum swinging formula is shown below [18]:

(1) $${\text{I}}_{\text{M}}{\text{θ}}^{\prime \prime }+\lambda \theta^{\prime }+mgl\theta =F$$

where ${\text{I}}_{\text{M}}$ is the system moment of inertia, m is the total mass of the pendulum, l is the effective distance between the swing axle and the center of the mass m, λ is the velocity factor of friction, g is gravity, and θ is the swing angle. The damping ratio of the two order system is derived as follows:

(2) $$\xi =\frac{\lambda }{\text{2}\sqrt{{\text{I}}_{\text{M}}mgl}}$$

It is found that almost all system parameters will affect the damping ratio ξ. Also, the resonant frequency will vary as the damping ratio ξ changes [6]. In this section, two proposed resonant cradle designs are described and evaluated. A cradle with free oscillation and low power consumption is the design goal. 

### 2.1. Resonant Cradle Design by Wind Force

**Figure 2:**
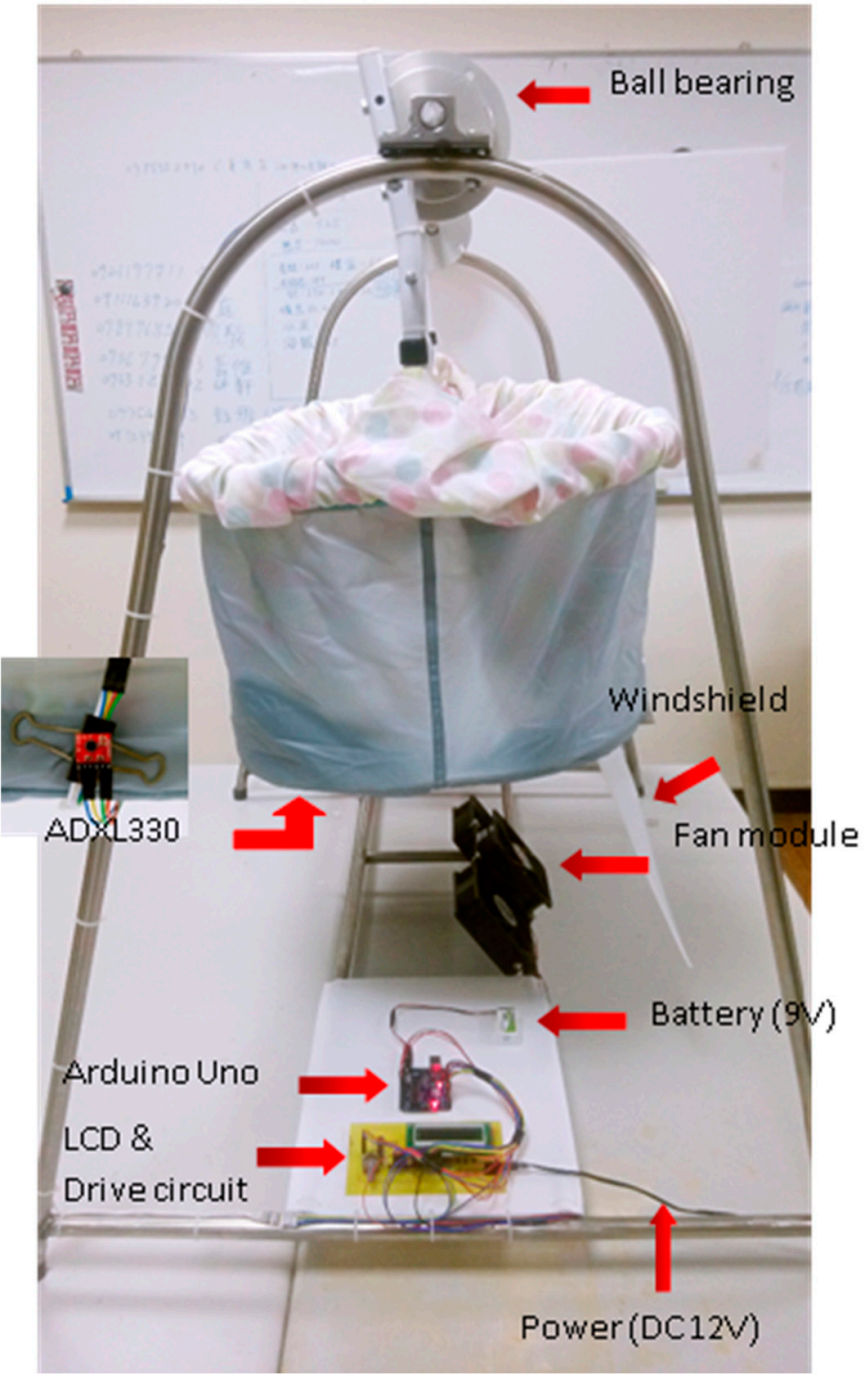
A resonant cradle design using wind power.

To make the swinging cradle an underdamped system with a smaller damping ratio, a ball bearing with little friction is placed on top of the cradle. Furthermore, to increase the distance from object to fulcrum and torque, the driving force is placed on the bottom of the cradle. Figure 2 shows the proposed first resonant cradle design, in which wind power is applied and a windshield board is installed to collect the wind power. This design can easily be moved manually without electricity. For example, if the user pulls the cradle to about a 15° swing angle and releases it, the cradle can sustain an oscillation for about 20 s, which is much longer than the above example of a commercial cradle design. 

An Arduino UNO board is programmed as the system controller, and is connected with a LCD & Drive circuit, which is composed of a LCD, buttons, and transistors (9013) for the driver of the fan module. The fan module contains three electric fans with an operating voltage of DC 12 V and an operating current of 0.7~0.83 A. An ADXL330 accelerometer is used to sense the swing angle and the ADC on the Arduino is set to use 10-bit (0~1023) to quantize the voltage output (0~3.3 V) of the ADXL330. 

The wind force was found to be inefficient in terms of power in a later performance evaluation. In addition, because the ADXL330 accelerometer swings with the cradle body and is installed below the cradle, the connecting wires must bypass the cradle by going up then down to the circuit board and they will swing with the cradle. In the next subsection, an improved resonant cradle design driven by motors with the blades below the cradle is proposed.

### 2.2. Resonant Cradle Design with Motor Driven Blades

**Figure 3:**
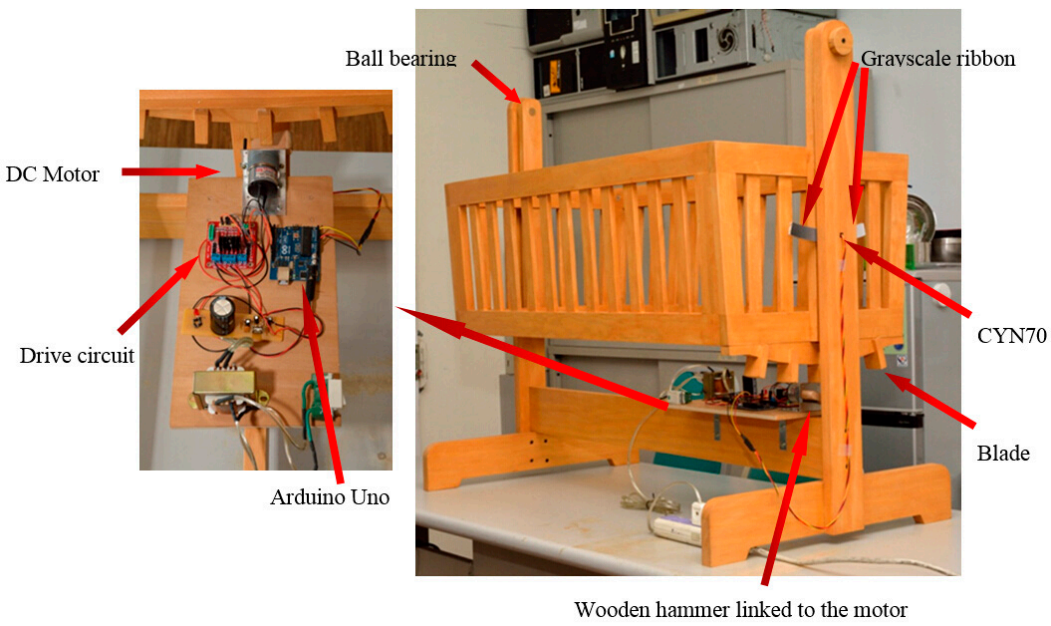
The proposed resonant cradle design with motor and blades.

The second proposed resonant cradle design is made of wood and is easy to assemble and disassemble as shown in Figure 3. A DC motor was installed under the cradle as the driving force, and the blades were spaced at a distance and tightly bounded under the cradle body. A small wooden hammer is linked to the motor to strike the blade and drive the cradle. The CNY70, a reflective sensor that includes an infrared emitter and phototransistor, was installed in a hole on the outer frame of the cradle and applied to sense the swing angle.

**Figure 4:**
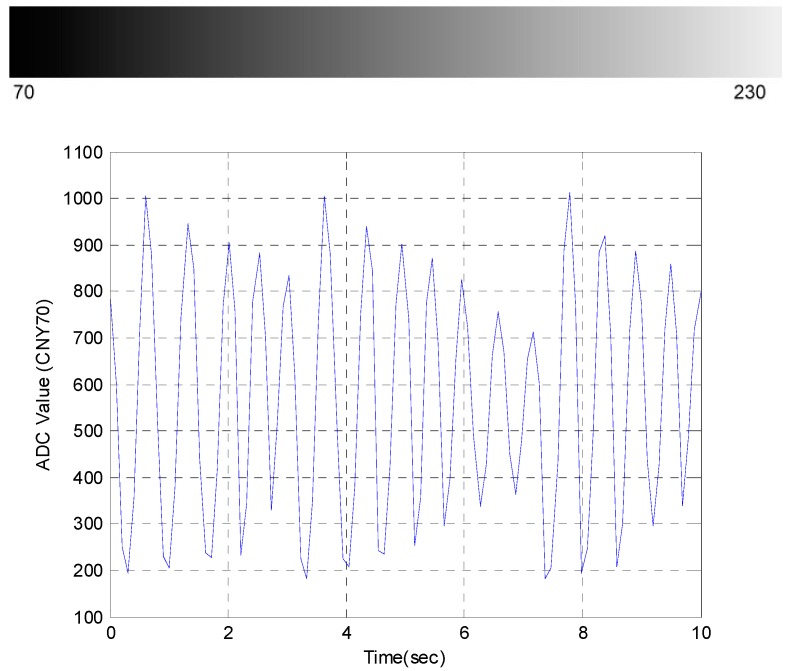
Grayscale ribbon and dynamic ADC value.

The grayscale quadrant shaped ribbon is on the rear side of the cradle and swings with the cradle. The ribbon was first printed with 70~230 gray levels, and then CNY 70 was used to sense the ribbon. Since gray levels 0–70 are unable to make the CNY 70 produce the voltage output, this non-sensitive region was abandoned. The distance between the head of CNY 70 and the ribbon is about 2 mm. When the cradle swings, the CNY 70 will sense the corresponding photosensitive value. Figure 4 shows the grayscale ribbon and the dynamic ADC value quantized by the Arduino UNO over 10 s. Based on the changes in these values, the swinging direction and angle can be detected and the controller will drive the blade, or the cradle, at the critical time. 

### 2.3. Performance Evaluation

Table 1 shows the performance evaluation based on the average power of the three design approaches. Although the wind powered design has the advantage of non-contact, experiments showed it to have low efficiency in terms of energy transfer because a windshield for collecting wind power is not the ideal design. Moreover, when the electric fan is on, it takes some time to reach the desired rotating speed and produce enough power. It will encounter the opposite situation when the fan is turned off. This will also influence the oscillation frequency so that the resonance characteristics cannot be exhibited clearly.

**Table 1:** Performance evaluation for average power.

Design Approach	Voltage	Average Current	Average Power
1. The wind powered resonant cradle design	12 V	1.63 A	19.56 W
2. The motor and blades resonant cradle design	9 V	Static: 0.115 A Dynamic: 0.129 A	Static: 1.035 W Dynamic: 1.161 W
3. The commercial design example	7 V	0.044 A	0.308 W

The average current and power measurement in the second resonant cradle design is divided into static and dynamic. In the static measurement, the power is on, but the swing angle is set to zero, so no motor operation occurs. The operating current of CNY 70 is only 1 mA, so at this moment, the power consumption is mainly for the Arduino UNO. In contrast, there is no micro-controller inside the commercial design example, so the power consumption is entirely for the motor operation. In the dynamic measurement of the second resonant cradle design, the motor is driven to sustain a fixed swing angle. According to experiments, to sustain a 15° swinging over 30 s only four motor stimulations are needed out of about 19 swing periods. 

**Figure 5:**
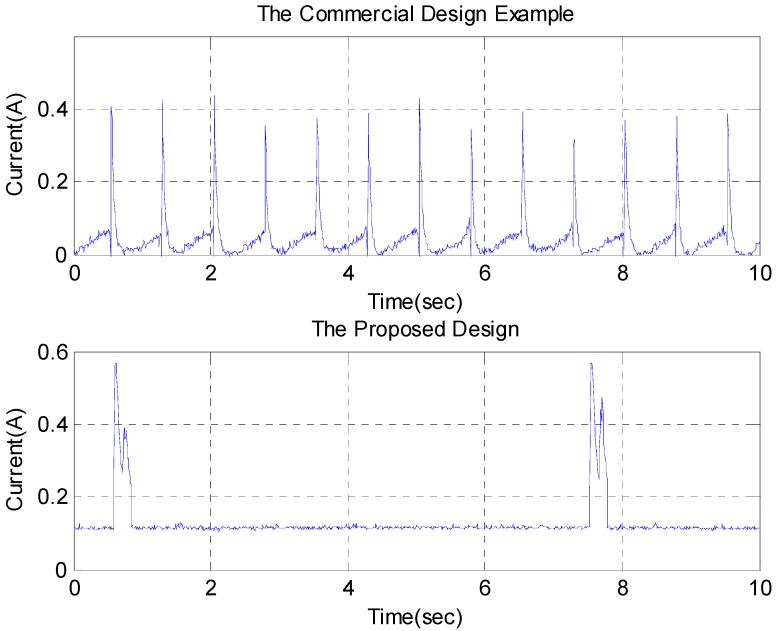
The waveforms of driving current: (**a**) the commercial design example; (**b**) the second resonant cradle design.

Figure 5 shows the Agilent DSO-X 2002A oscilloscope traces adopted to show the driving current waveform. In the commercial design example 1000 data points were recorded within 10 s (Figure 5a), indicating that the motor keeps rotating back and forth continually in this configuration. Figure 5b shows two motor stimulations with larger current pulses over 10 s for the second resonant cradle design. It seems reasonable to suggest that the average power consumption for the motor in the second resonant cradle design is the difference between the dynamic and static power measurement, that is, 1.161 W − 1.035 W = 0.126 W. If the motor efficiency problem [19,20] is omitted, it demonstrates that the second resonant cradle consumes less motor running power than the commercial design example. It is noted that the type number of the motor adopted in the second resonant cradle design is HN-35GBD-1634T, but the type number of the motor is unclear in the commercial design example.

**Figure 6:**
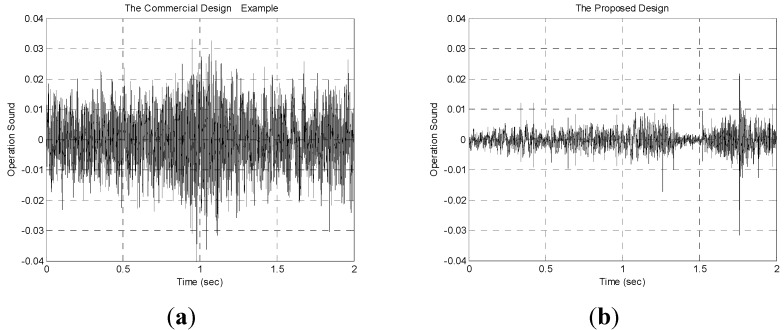
The waveforms of operation sound: (**a**) the commercial design example; (**b**) the second resonant cradle design.

The noise generated by the cradle is also another important factor in cradle design. In the commercial design example, the motor is on the top so its continuous operation combined with the bearing results in an annoying noise. However, in the proposed resonant cradle, there is almost no noise during free oscillation. When the wooden hammer strikes the blade in the second resonant cradle, it will cause an acceptable level of noise and the problem can be further improved by attaching a soft pad to the small wooden hammer. Figure 6 shows the waveforms of operating sound in the commercial design example and the second resonant cradle design. In Figure 6b, the sound pulse at about 1.75 s when the small wooden hammer strikes the blade.

Based on the above discussion, Table 2 summarizes and compares all the features of the commercial design example and the proposed second resonant cradle design. It shows the proposed second resonant cradle has several advantages over the commercial design.

**Table 2:** Feature comparison between the commercial design example and the proposed resonant cradle design.

	The Commercial Design Example	The Proposed Resonant Cradle
Damping system	Overdampled	Underdamped
Manually driven	Hard	Easy
Operation of motor	Rotates continually	Rotates only at critical time and consumes lower power
Cradle noise	Annoying	Low

## 3. Infant Cries Recognition

To provide infant cries recognition in an electric cradle, the first problem is to design the audio data acquisition circuit. In this section, infant cries acquisition is first described, followed by the proposed method to recognize the infant cries.

### 3.1. Infant Cries Acquisition

**Figure 7:**
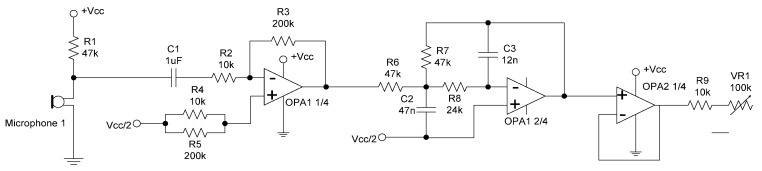
The pre-amplifier and anti-aliasing circuit.

The two stages of signal path from the microphone pickup to the analog input of Arduino UNO are shown in Figure 7 [21]. The signal from the microphone will go through an amplifier with a gain of 20, followed by an anti-aliasing low-pass filter using OPA134 with a cut-off frequency at 2 kHz. The technique of single-supply op-amp circuit, which is suitable for portable electronic equipment instead of a split-supply system, is applied [22] but there are also alternatives to implement the pre-amplifier and anti-aliasing circuit such as ISD1900, a multi message record/playback device [23]. 

**Figure 8:**
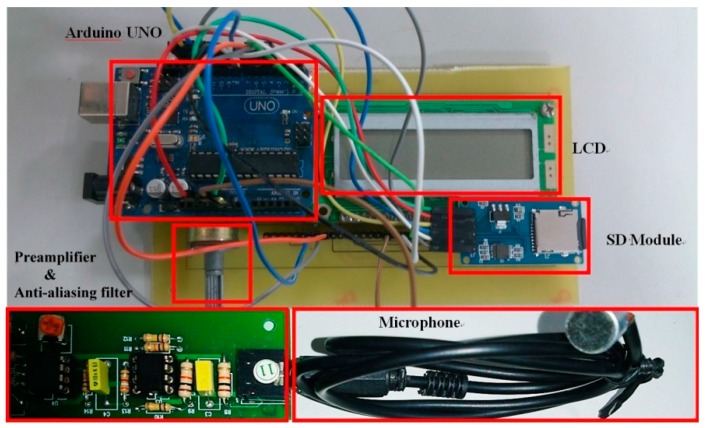
Hardware module prototypes.

The overall system hardware prototype is shown in Figure 8, including the Arduino UNO, the LCD and SD modules, pre-amplifier and anti-aliasing circuit, and the condenser microphone. With the SD module, the proposed design will allow the parents to record infant cries in the SD card, regardless of the limited resources of the Arduino UNO. The audio features are analyzed and recorded in the SD card for future recognition. The sampling rate is 8 kHz, and the bit resolution rate is 10 bits in the Arduino UNO.

**Figure 9:**
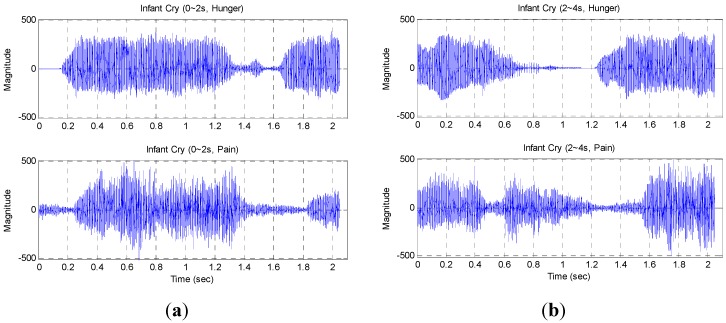
Infant cry waveforms: (**a**) about 0~2 s; (**b**) about 2~4 s.

The infant cries recognition is designed to increase the functionality of the proposed resonant cradle. The main feature is real-time processing by hardware implementation. Under such circumstances, the key point should be finding simple and efficient criteria to judge the audio features, instead of building a large database and adopting a complicated training methodology. Figure 9 shows the continuous infant hunger and pain cries waveforms from the same normal infant. Each recording lasts for about 2 s, or more precisely, it has an average of 2.048 s across 16,384 samples. The vertical value ranges from −512~511, which is constrained by quantized audio input ranging from 0~1023 by Arduino programming. It is worth mentioning that the paper only considers cries belonging to physiology because cries relating to pathology are beyond the scope of this paper.

### 3.2. Volume Intensity & Fundamental Frequency f0

In the hardware implementation, each recording is divided into frames for audio feature extraction. Each frame is arranged to have 256 samples, so there are 64 frames in all for a total of 16,384 samples in a recording. The sample number 256 and the frame number 64 are both values of a power of 2. It may have a faster performance under other possible FFT computations [24], but this is not elaborated on in this paper. 

The adopted Equation (3) measures the volume intensity for each frame with 10 times the 10-based logarithm of the sum of absolute samples x[n] within each frame. For almost all frames in Figure 9, the volume intensity is in the range of 30~50 dB. For the four plots in Figure 7, the values of average volume intensity are 42.27 dB (0~2 s, Hunger), 40.67 dB (0~2 s, Pain), 42.48 dB (2~4 s, Hunger), and 41.17 dB (2~4 s, Pain) sequentially.

(3) $$\text{E}=10\log_{10}\sum_{n}\left|x[n]\right|$$

Fundamental frequency f0 estimation is also referred to as pitch detection or extraction, and it can be accomplished in a frequency or in a time domain. In hardware design however, time domain should be more practical. There is a family of related time-domain f0 estimation methods which seek to discover how often the waveform fully repeats itself. The autocorrelation function (ACF) which (4), measures the correlation of a waveform with itself, is adopted to detect the f0:

(4) $$\text{R(}\nu {\text{)}}_{x}=\sum_{n=0}^{\infty }x[n]x[n-\nu ]$$

**Figure 10:**
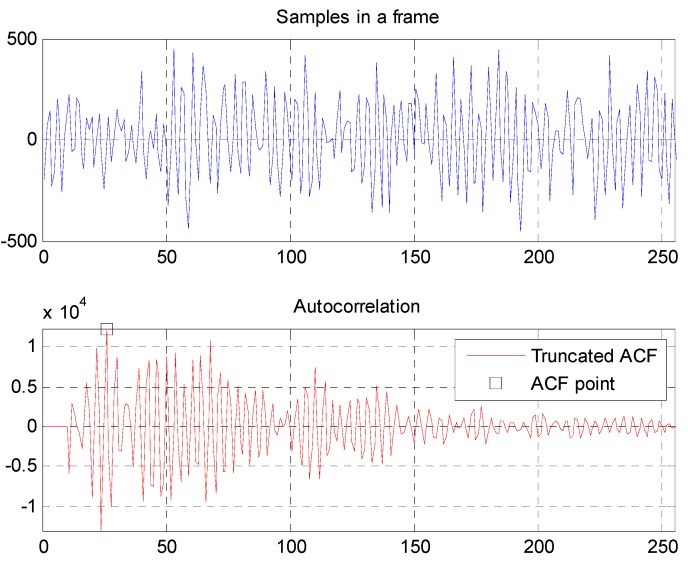
Autocorrelation of samples in a frame.

Figure 10 shows samples in a frame and the corresponding autocorrelation result. The calculation for f0 is shown in Equation (5), where $\nu_{\max}$ yields the maximum value in Equation (4). In Figure 10, $\nu_{\max}$ is 24 and f0 is 8000/23 = 347.8 Hz. The first ten outputs of ACF are truncated to zero to avoid invalid results [25]. It should be noted that samples in a frame with a volume intensity that is too low will also result in an invalid F0. For example, consider that all the samples in a frame are all zeros. In the proposed system, samples in a frame will be labelled as invalid when one of the following two conditions is satisfied:

1. The Volume Intensity is below 30 dB.
2. The calculated result of f0 is greater than 1000 Hz, or $\nu_{\max}\leq \text{9}$.

(5) $$f0=\frac{f_{s}}{\nu_{\max}-1}$$

### 3.3. Infant Cries Recognition

**Figure 11:**
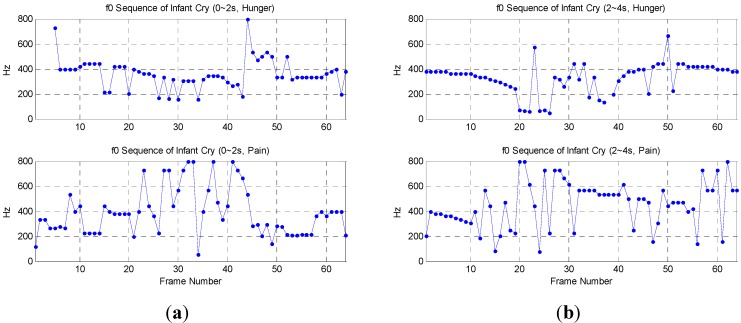
The f0 sequence of infant cries: (**a**) about 0~2 s; (**b**) about 2~4 s.

Figure 11 shows the f0 sequence of infant cries during about 0~2 s and 2~4 s respectively, each with 64 frames. It seems that cries of pain have higher f0 than cries of hunger. It should be noted that the first 4 points of the f0 sequence in the infant cries (0~2 s, Hunger) are labeled as invalid and cannot be plotted in Figure 11. 

The f0 sequence is shown as follows:

(6) $${\text{F0}}_{\text{s}}=\text{[}f0_{1},f0_{2},\cdots \cdots ,f0_{{\text{N}}_{\text{F}}}]$$

where $N_{F}$ is the number of frames, that is, $N_{F}$ equals 64. For each ${\text{F0}}_{\text{s}}$, it is desirable to obtain a synthesized result. The popular method is to take the average, which is expressed in Equation (7). It should be noted that the frames labeled as invalid will be excluded in all the following F0 analyses:

(7) $${\text{F0}}_{\text{Avg}}=\frac{1}{N_{F}}\sum_{i=1}^{N_{F}}f0_{i}$$

To emphasize the function of frames with stronger volume intensity, the proposed weighted average f0 analysis is shown in Equation (8). It is reasonable to assume that the frame with stronger volume intensity will have more impact on the final output of f0 analysis:

(8) $${\text{F0}}_{\text{WAvg}}=\frac{\sum_{i=1}^{{\text{N}}_{\text{F}}}f0_{i}\cdot E_{i}}{\sum_{\text{i}=\text{1}}^{{\text{N}}_{\text{F}}}{\text{E}}_{i}}$$

Both ${\text{F0}}_{\text{Avg}}$ and ${\text{F0}}_{\text{WAvg}}$ belong to linear operators, but to highlight the f0 changes, a nonlinear operator ${\text{F0}}_{\text{MAvg}}$ is developed and shown in Equation (9):

(9) $${\text{F0}}_{\text{MAvg}}=avg\{\max_{M}\{sort\{F0_{s}\}\}\}$$

where sort(⋅) is a function to sort sequence ${\text{F0}}_{\text{s}}$ from small to large, or large to small, $\max_{M}(\cdot )$ is a function to find the first M maximum values in a sequence, and avg(⋅) is a function to take the average. Table 3 shows the synthesized F0 analysis of different infant cries. The M value for $\max_{M}(\cdot )$ in ${\text{F0}}_{\text{MAvg}}$ is chosen as 15, which is acceptable for about a quarter of the ${\text{F0}}_{\text{s}}$ values with the highest fundamental frequencies.

**Table 3:** F0 analysis of infant cries.

F0 Analysis (Hz)	Hunger		Pain	
	0~2 s	2~4 s	0~2 s	2~4 s
${\text{F0}}_{\text{Avg}}$	356.63	369.17	397.36	468.98
${\text{F0}}_{\text{WAvg}}$	353.53	371.37	405.54	470.25
${\text{F0}}_{\text{MAvg}}$	507.03	456.80	678.87	684.23

Infant cries of pain indeed have higher f0 than cries of hunger, which is shown in Table 3. First, the ${\text{F0}}_{\text{Avg}}$ is applied, and then during 0~2 s, 356.63 Hz and 397.36 Hz will be set as the standard to distinguish between these two kinds of cries. However, the ${\text{F0}}_{\text{Avg}}$ of hunger increases to 369.17 Hz during 2~4 s, which is very close to the pain standard of 397.36 Hz and may lead to future misjudgments. The proposed ${\text{F0}}_{\text{WAvg}}$ is an improvement, but it is still insufficient. However, the ${\text{F0}}_{\text{MAvg}}$, a nonlinear operator, demonstrates it can effectively increase the frequency difference of F0 to about 200 Hz. 

**Figure 12:**
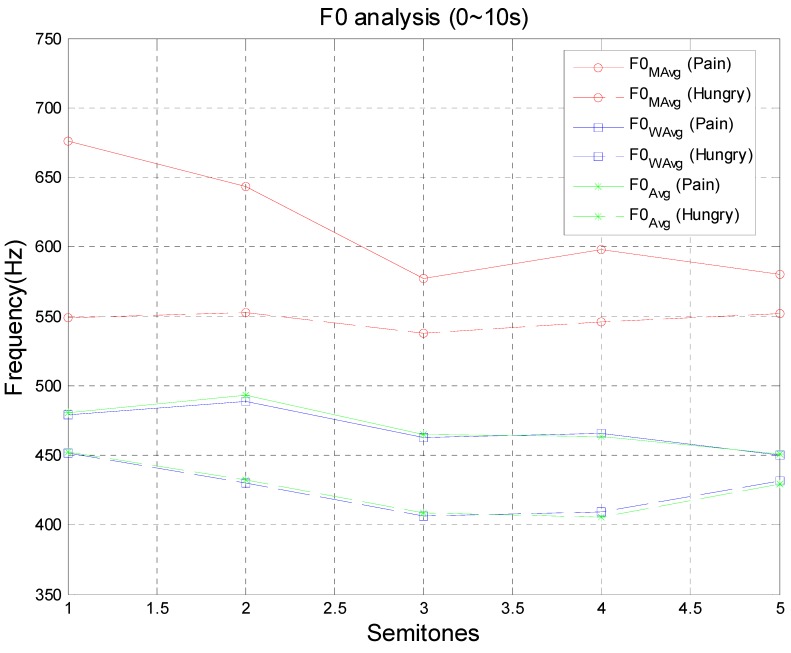
Plot of three different F0 analyses for infant cries.

To verify the efficiency of the ${\text{F0}}_{\text{MAvg}}$ analysis, another infant cry, lasting for 10 s, is recorded and tested. Figure 12 shows the F0 plot for three different analyses. It indicates ${\text{F0}}_{\text{WAvg}}$ has similar results to the ${\text{F0}}_{\text{Avg}}$, but ${\text{F0}}_{\text{MAvg}}$ has the ability to indicate the gap between cries of pain and cries of hunger making ${\text{F0}}_{\text{MAvg}}$ a useful analysis tool to enhance the recognition rate. 

Although past research showed no significant difference in fundamental frequency due to the sex or age of infant [16], recent research on fundamental frequency in the frequency domain has concluded that the male cry f0 (420 Hz) is higher than the female cry f0 (370 Hz) [26]. The proposed design will allow the users to record their infant cries of hunger or pain on an SD card and the proposed nonlinear operator ${\text{F0}}_{\text{MAvg}}$ it is expected to perform better for infant cries recognition.

## 4. Conclusions and Future Work

In this paper, an Arduino-based resonant cradle design with infant cries recognition was proposed. First, a ball bearing design is adopted to reduce system damping and let the cradle swing freely, even without electricity. Subsequently, an appropriate sensor is designed to detect the swinging status or angle. Finally, the force is put under the cradle to increase torque, but it engages only during a critical time. In other words, a small motor rotation angle is enough to make the cradle swing. The proposed design is an improvement on previous intelligent cradles as it naturally achieves the energy saving target in accordance with resonance theory. In addition, it has a much lower operating noise which will be welcomed by parents. 

Infant cries recognition provides inexperienced parents or babysitters with a reference for when an infant cries. With this function, the intelligent cradle can start swinging autonomously when the baby cries and stops when the swinging motion is no longer needed. The proposed design allows parents to record infant cries due to hunger or pain. Our experiment demonstrates the proposed nonlinear operator analysis in fundamental frequency is able to discriminate between the two kinds of infant cries making it superior to other intelligent cradle systems.

In the future, we hope to provide a mechanism to change the resonant frequency of the cradle, except the infant weight. The Arduino-based hardware design will ensure the diversity of future designs. Except for the adopted UNO, different versions with various capabilities, such as Mega, Leonardo, Yún, *etc*., have been developed. Furthermore, with Ethernet shield, an IP camera, and Webduino, real-time I/O monitoring and controlling in a browser can be accomplished quite easily. The idea of an Internet of Things will allow the cradle to connect to the Web and cloud to yield more innovative applications. It is also hoped that more infant cries can be used in the future, and, by connecting to the Internet, the recorded cries will be available for medical diagnosis. Some modern techniques, such as pattern recognition, will be applied to recognize more types of infant cries in the aspect of intelligence.
